# Supplementary material for: Chemotherapy-driven intestinal dysbiosis and indole-3-propionic acid rewire myelopoiesis to promote a metastasis-refractory state
Source: Nat Commun. 2025 Dec 15;17:832. doi: 10.1038/s41467-025-67169-7 (PMC12827274; doi:10.1038/s41467-025-67169-7)
Supplement: Supplementary file 1 — Supplementary Information [file 41467_2025_67169_MOESM1_ESM.pdf]

Supplementary Information

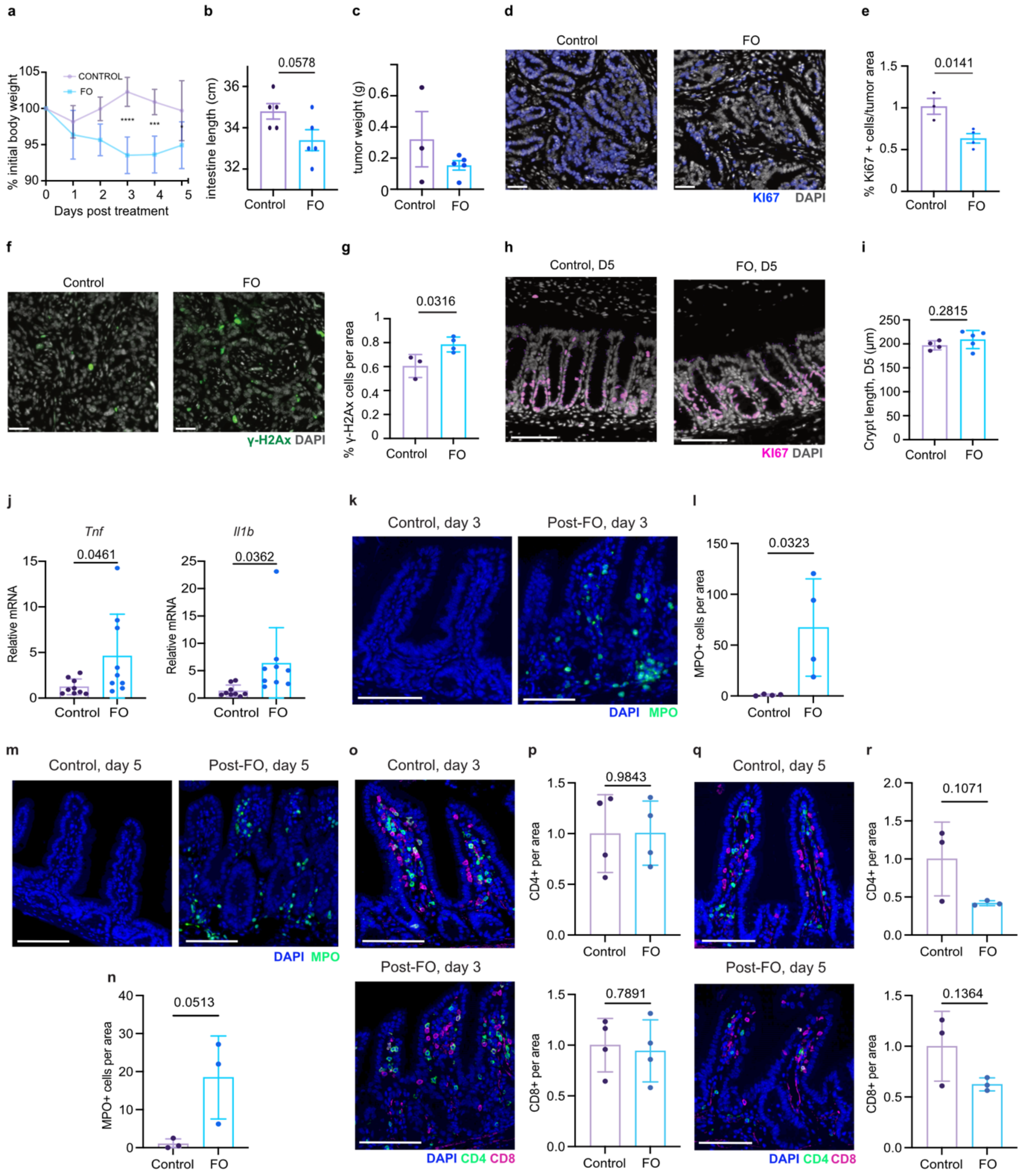


**Supplementary Figure 1 related to Figure 1. 5-FU + oxaliplatin (FO) induce intestinal lesions and effective antitumor effect in murine model.**

(**a**) Percentage of initial body weight and (**b**) intestinal length in cm at day three post FO. n = 5 per condition. (**c**) Tumor weights five days after treatment. Control, n=3; FO, n=5. (**d**) FO reduces tumor cell proliferation. Staining for KI67 (blue) and DAPI (grey). Scale bars: 100μm. (**e**) Percentage of KI67 positive nuclei per tumor area normalised to control. Control, n = 3; FO, n = 4. (**f**) Staining for γ-H2Ax (green) and DAPI (grey). Scale bars: 100μm. (**g**) Quantification of cancer cell DNA damage. Percentage of γ-H2Ax positive cells. Control, n =

3; FO, n = 4. (**h**) Colon sections five days post FO, KI67 (magenta) and DAPI (grey). Scale bars: 100μm. (**i**) Quantification of crypt length in μm. Control, n = 4; FO, n = 5. (**j**) qPCR analysis of *Tnf* and *Il1b* in the ileum of mice 5 days after treatment with control (PBS) or FO. Data are normalized to housekeeping gene *18S*. Data from two independent experiments. n = 9 per condition. (**k-l**) MPO+ cells infiltrate the ileum of FO treated mice 3 days post-treatment. Images show MPO (green) and DAPI (blue). Scale bars: 100μm. Quantification MPO+ cells normalized to area. n = 4 per condition. (**m-n**) MPO+ cells infiltrate the ileum of FO treated mice 5 days post-treatment. Images show MPO (green) and DAPI (blue). Scale bars: 100μm. Quantification MPO+ cells normalized to area. n = 3 per condition. (**o-p**) Images show CD4 (green), CD8 (magenta) and DAPI (blue). Quantification CD4+ and CD8+ cells normalized to area and control mean. n = 4 per condition. Scale bars: 100μm **(q-r**) CD4+ and CD8+ cell infiltration are similar 5 days post-treatment. Images show CD4 (green), CD8 (magenta) and DAPI (blue). Quantification CD4+ and CD8+ cells normalized to area and control mean. n = 3 per condition. Scale bars: 100μm. Data are shown as mean ± SD and analyzed using two-tailed unpaired Student's t-test (**b, c, e, g, i, j, l, n, p, r**).

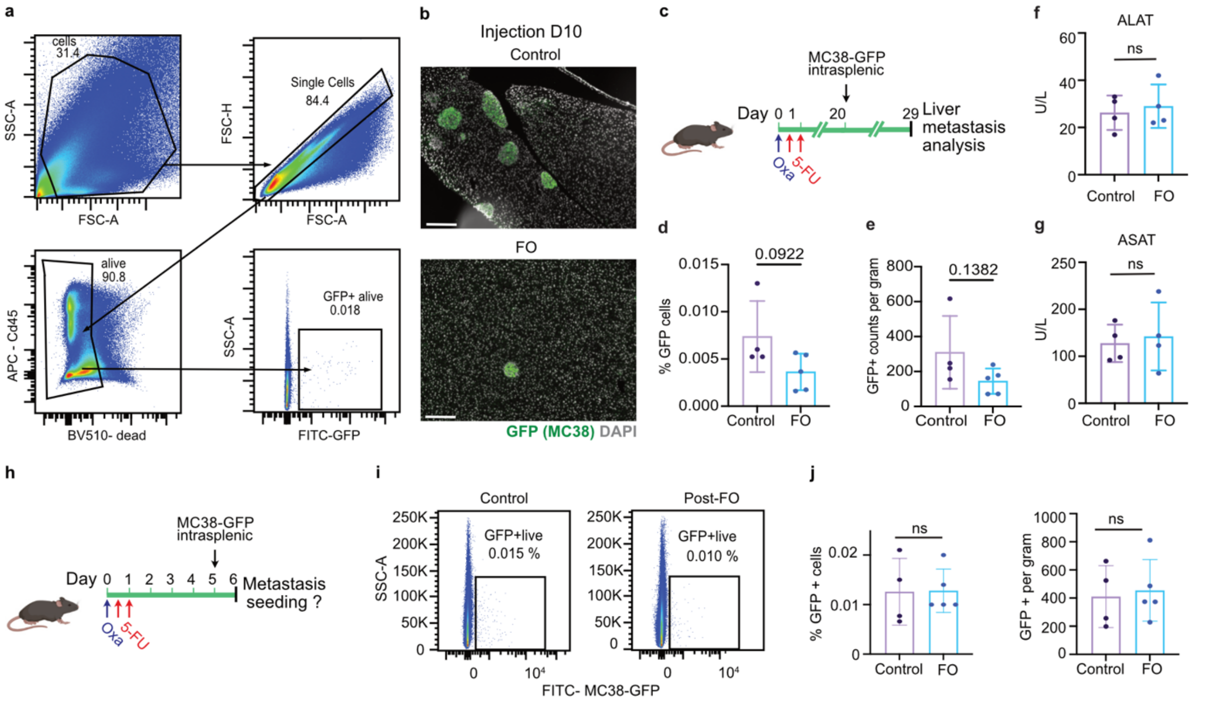


**Supplementary Figure 2 related to Figure 1. FO maintained a lasting chemomemory effect.**

(**a**) Gating strategy for MC38-GFP+ tumor cell detection in the liver. (**b**) FO preconditioning reduces liver metastasis formation by MC38-GFP tumor cells injected ten days after FO. Staining for MC38-GFP tumor cells (green) and DNA (grey). Scale bar: 100µm. (**c**) Scheme of the experiment. MC38-GFP tumor cells were injected intrasplenically 20 days after FO preconditioning. The livers were harvested 9-12 days after tumor cell injection. (**d**) Quantification of percentage and (**e**) count of GFP positive cancer cells in the indicated conditions. Control, n = 4. FO, n = 5. **(f-g**) FO does not induce direct hepatotoxicity. (**f**) Alanine-aminotransaminase (ALAT) and (**g**) aspartate-aminotransferase (ASAT) serum concentration in control and FO treated mice. n = 4 per condition. (**h**) Scheme of the experiment. (**i**) FO preconditioning does not impact survival of cancer cells in circulation or metastatic seeding. Representative FACS plots of GFP+ cancer cells in livers of control and 5 days post-FO mice, analyzed 24h after tumor cell injection. (**j**) Quantification of percentage and count of GFP positive cancer cells in the indicated conditions. Control, n = 4. FO, n = 5. Data are shown as mean ± SD and analyzed using two-tailed unpaired Student’s t-test (**d, e, f, g, j**). Icons were created in *BioRender under the license SABINE, A. (2025)* *https://BioRender.com/ pmy8rs7*.

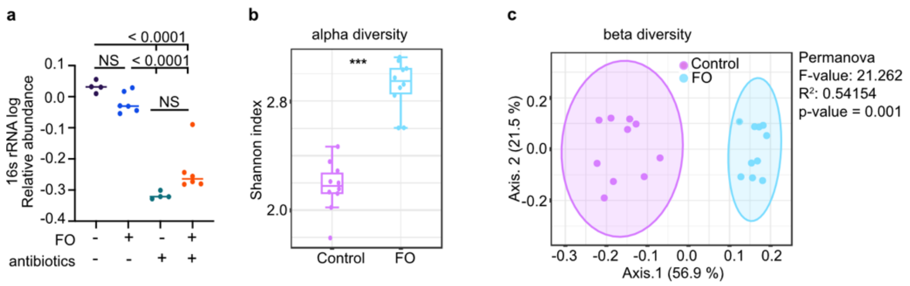


**Supplementary Figure 3 related to Figure 2. Chemotherapy modifies gut microbiota composition.**

(**a**) Bacterial 16s rRNA quantification of metastasis experiment by qPCR. (**b**) Alpha diversity analysis showing increase Shannon index in FO treated mice. P-value was calculated using Welch T-Test. P-value ***<0.0001. (**c**) Principal coordinate analysis (PCoA) of microbiota from mice stool 5 days after FO or PBS treatment using Bray-Curtis distances. Data are shown as mean ± SD and analyzed one-way Anova with Tukey's multiple comparisons test (**a**).

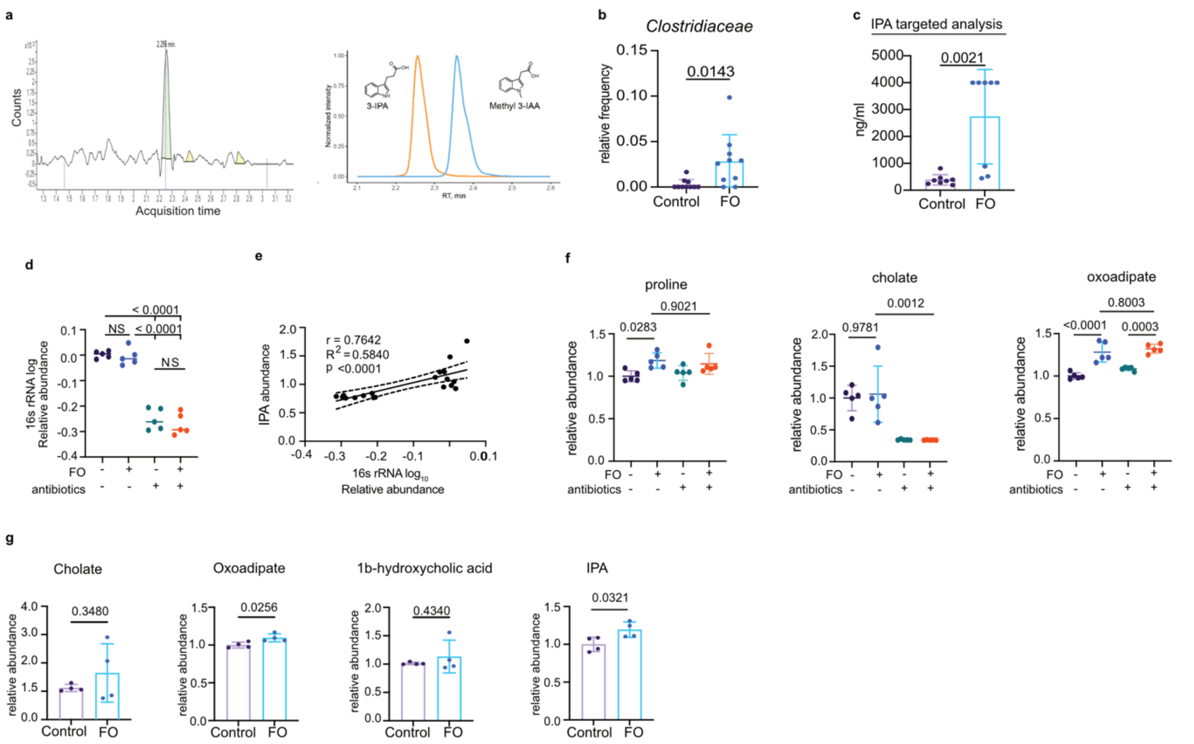


**Supplementary Figure 4 related to Figure 3. FO induces a lasting metabolome change in portal serum.**

(**a**) IPA identity confirmation by retention time. (**b**) Relative frequency of Clostridiaceae, n = 10 per conditions from the 16s rRNA sequencing. (**c**) IPA concentration measured by LC-MS/MS, 5 samples from FO-treated mice reached upper detection limit. n = 8 per condition. (**d**) Bacterial 16s rRNA quantification of metabolomics experiment by qPCR. (**e**) Correlation between IPA relative abundance and bacterial 16s rRNA abundance. n = 5 per condition. (**f**) Relative abundance of metabolites increased upon FO in microbiota depleted mice after 5 days FO. n = 5 per condition. (**g**) Relative abundance of metabolites in portal serum sampled ten days after FO or PBS. Data are shown as mean ± SD and analyzed using two-tailed unpaired Student's t-test (**b, c**) with multiple testing correction (**g**), Pearson correlation test (**e**), one-way Anova with Tukey's multiple comparisons test (**d, f**).

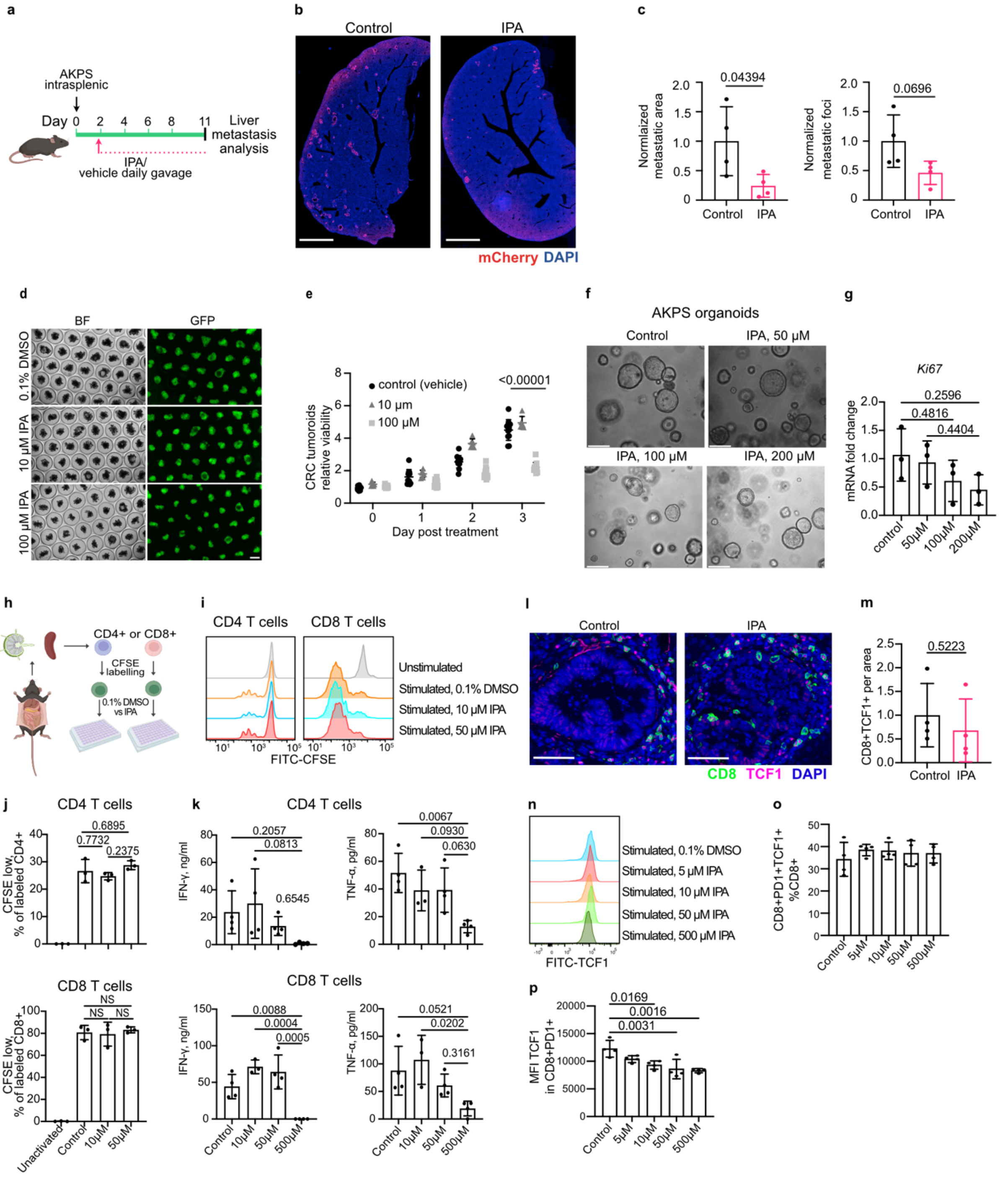


**Supplementary Figure 5 related to Figure 4. IPA does not directly affect cancer cells or CD4+ and CD8+ T cell antitumor activity.**

(**a**) Scheme of the experiment. (**b**) IPA inhibits liver metastasis growth. Staining for mCherry (red) and DNA (blue). Scale bar: 2mm. (**c**) Quantification of metastasis area and number. Data normalized to liver area and control mean. n = 4 per condition. (**d**) Luciferase-expressing human CRC tumoroids were treated with IPA or control (DMSO). Scale bar, 300 μm. (**e**) Quantification of CRC viability, assessed by luciferase signal. Each dot represents individual mouse or independent in vitro experiment. (**f**) Representative images of AKPS organoids exposed to indicated concentration of IPA or vehicle (DMSO). Two independent experiments. Scale bar: 100μm. (**g**) mRNA level of *Mki67* normalized to 18S RNA expression levels. n = 3

per condition. (**h**) Scheme of the experiment to analyze the effect of IPA on CD4+ and CD8+ T cells. (**i**) IPA does not affect proliferation of activated CD4+ and CD8+ T cells in vitro. Histogram of CFSE dilution in CD4+ (left) and CD8+ (right) cells. (**j**) Quantification of CD4+ and CD8+ proliferated cells in the indicated conditions. n = 3 per condition. (**k)** IFNγ and TNFα levels in cell culture supernatant three days after exposure. n = 3 to 4 biological replicates per condition. (**l**) Representative image of CD8+TCF1+ cells in metastatic lesions. CD8 (green), TCF1 (magenta) and DNA (blue). Scale bar: 50µm. (**m**) IPA does not change peritumoral CD8+TCF1+ T cell infiltration. Quantification of CD8+TCF1+ cells per metastatic area. n = 4 per condition. (**n**) IPA does not increase CD8+ exhausted progenitor cells. Histogram of TCF1 expression in CD8+PD1+ T cells. (**o**) Quantification of the percentage of CD8+PD1+TCF1+ cells. (**p**) Quantification of TCF1 MFI in CD8+PD1+ T cells. (**o-p**) n = 4 per condition. Data are shown as mean ± SD and analyzed using two-tailed unpaired Student's t-test (**c, m**) and one-way Anova with Tukey's multiple comparisons test (**e, g, j, k, o, p**). Icons were created in *BioRender under the license SABINE, A. (2025) https://BioRender.com/ pmy8rs7*.

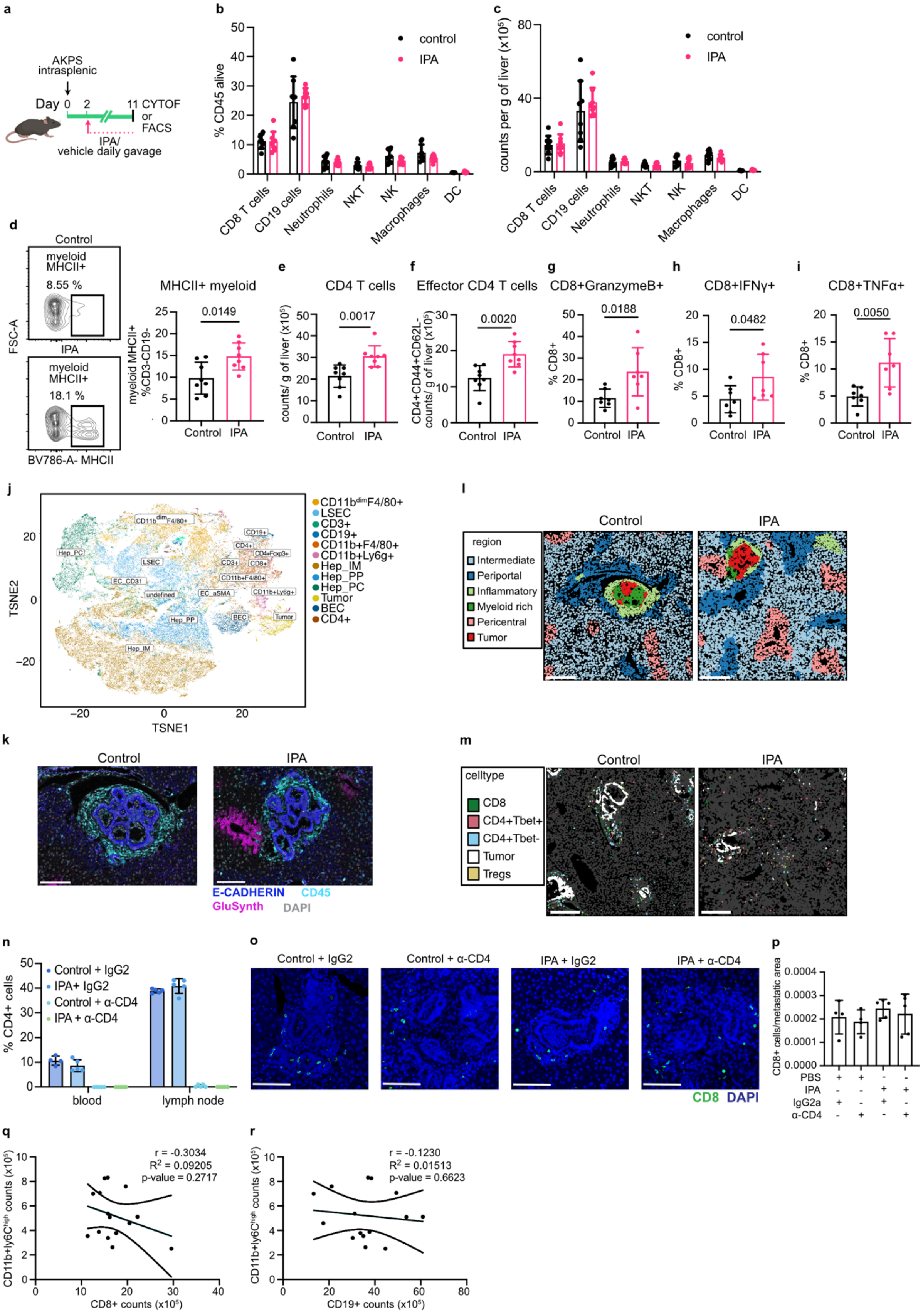
a
AKPS intrasplenic
Day 0 2 11 CYTOF or FACS
IPA/ vehicle daily gavage
b
% CD45 alive
control
IPA
CD8 T cells
CD19 cells
Neutrophils
NKT
NK
Macrophages
DC
c
counts per g of liver (x10^5)
control
IPA
d
Control
myeloid MHCII+
8.55 %
IPA
myeloid MHCII+
18.1 %
FSC-A
BV786-A- MHCII
MHCII+ myeloid
myeloid MHCII+ %CD3-CD19-
0.0149
Control IPA
e
CD4 T cells
counts/ g of liver (x10^5)
0.0017
Control IPA
f
Effector CD4 T cells
CD4+CD44+CD62L- counts/ g of liver (x10^5)
0.0020
Control IPA
g
CD8+GranzymeB+
% CD8+
0.0188
Control IPA
h
CD8+IFNγ+
% CD8+
0.0482
Control IPA
i
CD8+TNFα+
% CD8+
0.0050
Control IPA
j
TSNE2
TSNE1
CD11b^dim F4/80+
LSEC
CD3+
CD19+
CD11b+F4/80+
CD11b+Ly6g+
Hep_IM
Hep_PP
Hep_PC
Tumor
BEC
CD4+
l
Control
IPA
region
Intermediate
Periportal
Inflammatory
Myeloid rich
Pericentral
Tumor
k
Control
IPA
E-CADHERIN CD45
GluSynth DAPI
m
Control
IPA
celltype
CD8
CD4+Tbet+
CD4+Tbet-
Tumor
Tregs
n
% CD4+ cells
Control + IgG2
IPA+ IgG2
Control + α-CD4
IPA + α-CD4
blood
lymph node
o
Control + IgG2
Control + α-CD4
IPA + IgG2
IPA + α-CD4
CD8 DAPI
p
CD8+ cells/metastatic area
PBS
IPA
IgG2a
α-CD4
q
r = -0.3034
R² = 0.09205
p-value = 0.2717
CD11b+ly6C^high counts (x10^5)
CD8+ counts (x10^5)
r
r = -0.1230
R² = 0.01513
p-value = 0.6623
CD11b+ly6C^high counts (x10^5)
CD19+ counts (x10^5)

**Supplementary Figure 6 related to Figure 5. IPA modifies the immune cell composition of the metastatic liver.**

(**a**) Scheme of the experiment to analyze the IPA effect on the tumor immune microenvironment. (**b-c**) Immune cell composition of metastatic liver in control and IPA treated mice. (**b**) Data show percentage of total CD45+ cells. (**c**) Data show counts per gram of liver. n = 8 per condition. (**d**) IPA increases accumulation of myeloid MHCII+ cells in metastatic liver. n = 8 per condition. Data are from two independent experiments. (**e**) Counts of CD4+ T cells. (**f**) Counts of effector CD4 (CD44+CD62L-) T cells in the metastatic liver. n = 8 per condition. (**g**) Percentage of CD8+ granzyme b (GZMB) +, (**h**) CD8+ IFNγ and (**i**) CD8+TNFα. n = 7 per condition. (**j**) Mass cytometry imaging T-SNE plot showing the isolated cell populations. (**k**) Mass cytometry imaging showing CD45+ infiltrate around metastatic lesion. Staining for E-cadherin (blue), CD45 (cyan), glutamate synthase (GluSynth, magenta) and DNA (white). Scale bar: 100µm. (**l**) Local spatial autocorrelation analysis showing regions spatial distribution. (**m**) Masked plot showing CD4+Tbet+ (red), CD4+Tbet- (blue), CD4+FOXP3+ (yellow), CD8+ (green) and tumor (white) spatial distribution. Scale bar: 200µm. (**n**) Percentage of CD4+ cells in blood and lymph nodes of control IgG2 or α-CD4 treated mice. (**o**) Representative image of CD8+ infiltration in metastatic lesions. Staining for CD8 (green) and DNA (blue). Scale bar: 100µm. (**p**) Quantification of CD8 cells per metastatic area. Control + IgG2a, n = 4. Control + α-CD4, n = 4. IPA + IgG2a, n = 5. IPA + α-CD4, n = 5. (**q**) Pearson correlation of liver Ly6C$^{high}$ monocytes and CD8+ counts. (**r**) Pearson correlation of liver Ly6C$^{high}$ monocyte and CD19 counts. **(q-r)** n = 8 per condition. Data are shown as mean ± SD and analyzed using one-way Anova with Tukey's multiple comparisons test (**b, c, p**), two-tailed unpaired Student's t-test (**d, e, f, g, h, i**) and Pearson correlation (**q, r**). Icons were created in *BioRender under the license SABINE, A. (2025) https://BioRender.com/ pmy8rs7*.

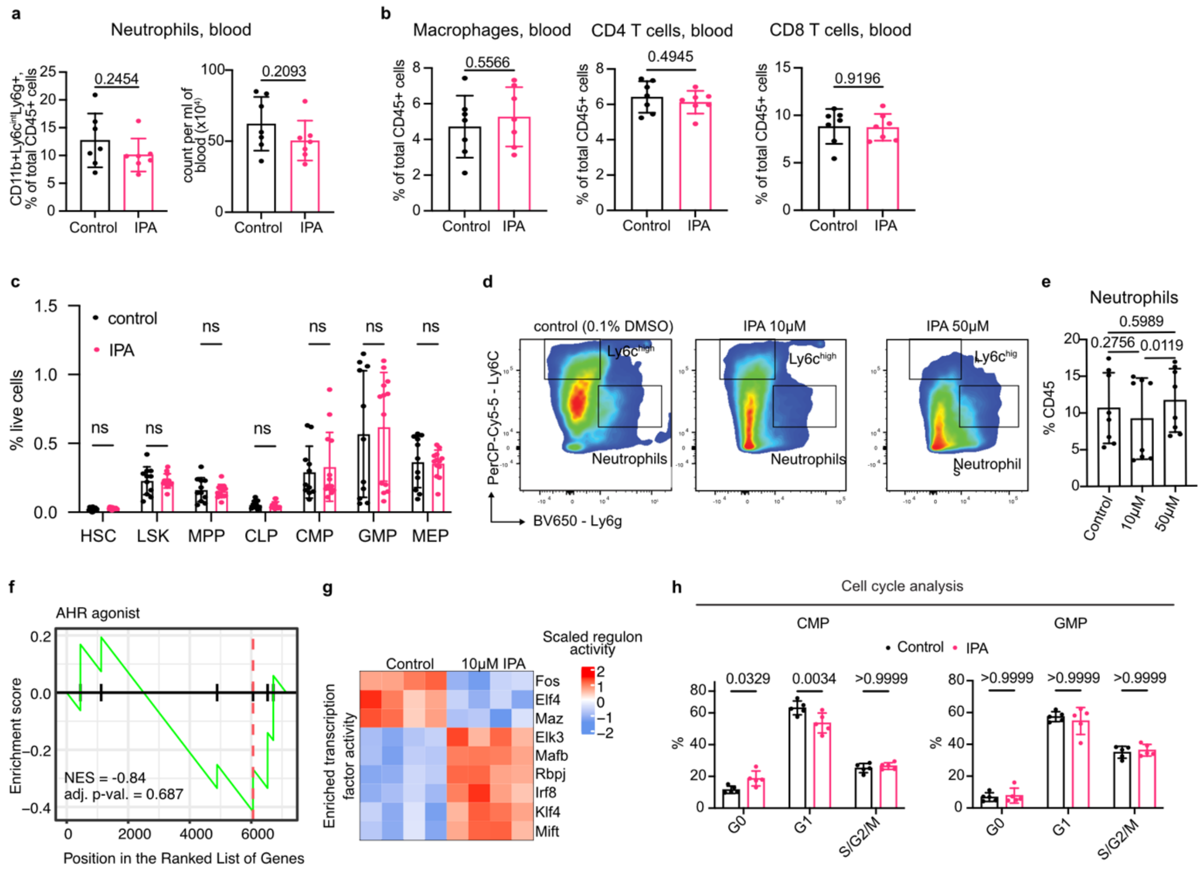

**Supplementary Figure 7 related to Figure 6. IPA affects myeloid cell differentiation without affecting progenitor numbers.**

(**a-b**) IPA does not influence neutrophils, macrophages, CD4+ and CD8+ T cell abundance in the blood. n = 7 per conditions (**c**) IPA does not influence bone marrow progenitor abundance. Data shows percentage of cells in the bone marrow of mice gavage with PBS or IPA. Control, n = 11; IPA, n = 13. Pooled from two independent experiments. (**d**) IPA inhibits generation of Ly6C$^{high}$ monocytes but not neutrophils *in vitro*. FACS plot showing reduced Ly6C$^{high}$ monocytes. (**e**) Percentage of neutrophils generated with vehicle (DMSO) or IPA in vitro. n = 8 per condition. Data show biological replicate pooled from two independent experiments. (**f**) AHR gene signature is not modified by IPA in bone marrow cells. (**g**) SCENIC transcriptional network analysis shows that IPA activates regulons for myeloid cell maturation/differentiation. (**h**) CMP (Lineage$^{-}$cKit$^{+}$Sca1$^{-}$(KLS$^{-}$ )CD16/32$^{low}$CD34$^{+}$) in IPA treated mice are more quiescent. Control, n = 5. IPA, n = 5. GMP (KLS$^{-}$CD16/32$^{low}$CD34$^{+}$). Data are shown as mean ± SD and analyzed using two-tailed unpaired Student's t-test (**a, b),** one-way Anova with Tukey's multiple comparisons test (**c**), repeated measure one-way Anova with Tukey's multiple comparisons test (**e**) and two-way Anova with Bonferroni's multiple comparisons test (**h**).

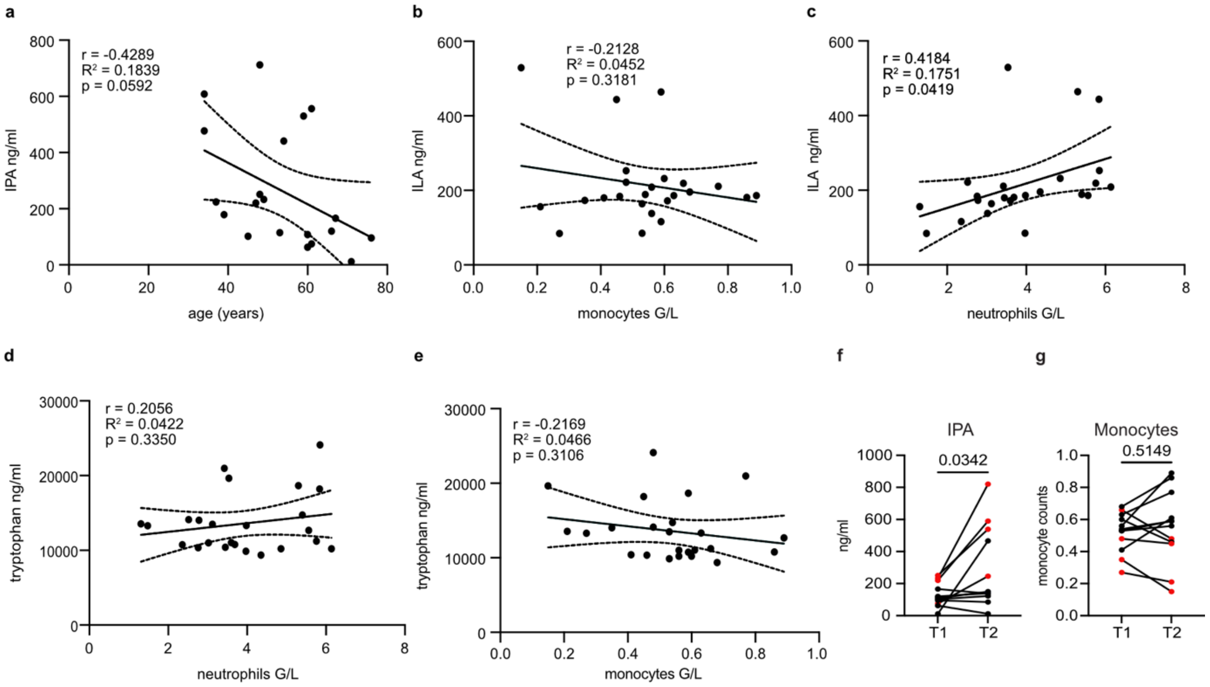


**Supplementary Figure 8 related to Figure 8. Further targeted metabolomics analyses of blood from chemonaive CRC patients.**

(**a**) IPA concentration in blood does not correlate with age in healthy and CRC patient cohorts. n = 8 healthy controls and 12 CRC patients. (**b**) ILA concentration does not correlate with monocytes and (**c**) neutrophils abundance in CRC patients. (**d**) Tryptophan concentration does not correlate with neutrophils and (**e**) monocytes abundance in CRC patients. (**f**) and (**g**) Highlighted in red are the patients with the IPA increase and monocytes reduction at T2. Data are shown as mean ± SD, and analyzed using Pearson correlation test (**a, b, c, d, e**), two-tailed Wilcoxon rank-sum test (**f**) and two-tailed paired Student's t-test (**g**).

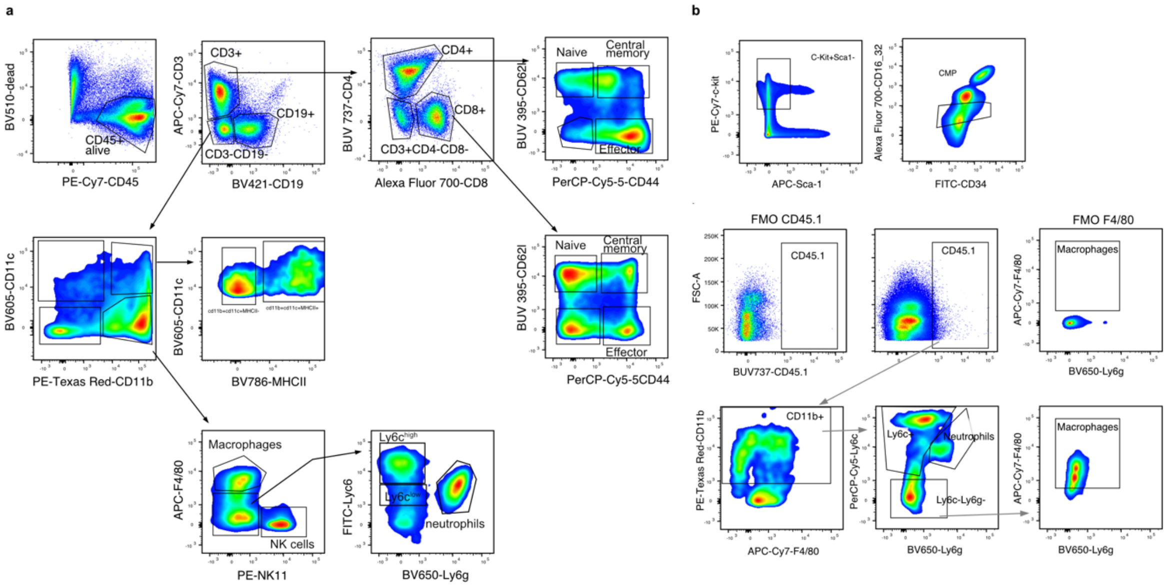


**Supplementary Figure 9. Gating strategy used in the manuscript.**
(a) Gating strategy related to Fig. 5 and Supplementary Fig. 6. (**b**) Gating startegy related to Fig. 6j-l and supplementary Fig. 7.

## Supplementary Table 1. CRCCT and SwissChronoFood cohorts.

| **CRCCT cohort** | | | **SwissChronoFood** | | |
|---|---|---|---|---|---|
| Age, years | | | Age, years | | |
| | 30-59, no (%) | 6 (42.85%) | | 30-59, no (%) | 7 (87.5%) |
| | 60-79, no (%) | 8 (57.14%) | | 60-79, no (%) | 1 (12.5%) |
| Sex | | | Sex | | |
| | male, no (%) | 8 (57.14%) | | male, no (%) | 6 (75%) |
| | Female, no (%) | 6 (42.85%) | | Female, no (%) | 2 (25%) |
| MSI/MSS | | | antibiotics > 4 weeks before | | |
| | MSI, no (%) | 3 (21.42%) | | no, no (%) | 8 (100%) |
| | MSS, n (%) | 10 (71.42%) | | | |
| | undetermined, no (%) | 1 (7.14%) | | | |
| Tumor pathological stage | | | | | |
| | IIa, no (%) | 1 (7.14%) | | | |
| | IIIa, no (%) | 1 (7.14%) | | | |
| | IIIb, no (%) | 3 (21.42%) | | | |
| | IIIc, no (%) | 5 (35.71%) | | | |
| | IV, no (%) | 4 (28.57%) | | | |
| Chemotherapy | | | | | |
| | CAPOX, no (%) | 9 (64.28%) | | | |
| | FOLFOX, no (%) | 4 (28.57%) | | | |
| | Capecitabine, no (%) | 1 (7.14%) | | | |
| Targeted therapy | | | | | |
| | Bevacizumab, no (%) | 1 (7.14%) | | | |
| antibiotics > 4 weeks before | | | | | |
| | yes, no (%) | 12 (85.71%) | | | |
| | no, no (%) | 2 (14.28%) | | | |

## Supplementary Table 2. The list of primers used in the study

| | Forward | Reverse | References |
|---|---|---|---|
| *18s* | 5'- AGGAATTCCCAGTAAGTGCG-3' | 5'- GCCTCACTAAACCATCCAA-3' | https://pubmed.ncbi.nlm.nih.gov/26389677/ |
| *16s* | 5′-*CGGTGAATACGTTCCCGG* -3′ | 5′-TACGGCTACCTTGTTACGACTT-3′ | this paper |
| *Tnf* | 5'- TGGAAGTAGACAAGGTACAACCC -'3 | 5'- CATCTTCTCAAAATTCGAGTGACAA-3' | https://doi.org/10.1084/jem.20212418 |
| *Il1b* | 5'-CAACCAACAAGTGATATT CTCCATG-3' | 5' GATCCACACTCTCCAGCTGCA-3' | https://doi.org/10.1084/jem.20212418 |
| *Mki67* | 5'-ATCATTGACCGCTCCTTTAGGT-3' | 5'-GCTCGCCTTGATGGTTCCT-3' | this paper |
