# Supplementary material for: Chemotherapy-driven intestinal dysbiosis and indole-3-propionic acid rewire myelopoiesis to promote a metastasis-refractory state
Source: Nat Commun. 2025 Dec 15;17:832. doi: 10.1038/s41467-025-67169-7 (PMC12827274; doi:10.1038/s41467-025-67169-7)
Supplement: Supplementary file 2 — Description of Additional Supplementary Files [file 41467_2025_67169_MOESM2_ESM.pdf]

Title: Supplementary Data 1

Description: metabolite intensities untargeted metabolomics in the ileum, portal blood of mice treated with control or FO

Title: Supplementary Data 2

Description: log2(FC) abundance of metabolite in the cecal content of control versus FO treated mice

Title: Supplementary Data 3

Description: metabolite intensities in the blood of mice treated with FO or control +/- antibiotics

Title: Supplementary Data 4

Description: CYTOF imaging cell type population

Title: Supplementary Data 5

Description: Related to figure 6.Genes differentially expressed in BM cells treated in MDSC inducing medium, control versus 10uM IPA; Differential gene expression analysis was performed using DESeq2

Title: Supplementary Data 6

Description: gradient and injection in matbolon facility
